# Optical and Electronic Properties of Femtosecond Laser-Induced Sulfur-Hyperdoped Silicon N+/P Photodiodes

**DOI:** 10.1186/s11671-017-2287-2

**Published:** 2017-09-04

**Authors:** Ting Zhang, Bohan Liu, Waseem Ahmad, Yaoyu Xuan, Xiangxiao Ying, Zhijun Liu, Zhi Chen, Shibin Li

**Affiliations:** 1 School of Optoelectronic Information, University of Electronic Science and Technology of China, Chengdu, Sichuan 610054 China; 2 Department of Electrical and Computer Engineering, University of Kentucky, Lexington, KY 40506 USA; 3 Center for Nanoscale Science and Engineering, University of Kentucky, Lexington, KY USA

**Keywords:** Ion-implantation, Hyperdoped silicon, NIR photoresponse

## Abstract

Impurity-mediated near-infrared (NIR) photoresponse in silicon is of great interest for photovoltaics and photodetectors. In this paper, we have fabricated a series of n^+^/p photodetectors with hyperdoped silicon prepared by ion-implantation and femtosecond pulsed laser. These devices showed a remarkable enhancement on absorption and photoresponse at NIR wavelengths. The device fabricated with implantation dose of 10^14^ ions/cm^2^ has exhibited the best performance. The proposed method offers an approach to fabricate low-cost broadband silicon-based photodetectors.

## Background

Traditional silicon-based devices could not show desirable NIR photoresponse due to limitation of optical bandgap (1.12 eV) of silicon [1], and many attempts have been made to enhance the absorptance of silicon material, especially at NIR wavelengths [2–9]. The discovery of chalcogen-supersaturated silicon fabricated by laser irradiation in SF_6_ atmosphere demonstrated an approach to enhance the sub-bandgap absorption [10, 11]. In this process, the material can be doped beyond the solubility limit [12]. Besides, light trapping effect caused by the unique pointed cone structure on silicon surface also increases the efficiency of light absorption [13]. In this paper, we have fabricated hyperdoped silicon prepared by ion-implantation and femtosecond pulsed laser. Hall measurement was carried out to measure the electrical properties of hyperdoped silicon. Photodetectors based on n^+^/p junction demonstrated high performances on both NIR absorption and photoresponse.

## Methods

One-side polished p-type silicon [100] wafers (300 μm) with resistivity 8–12 Ω cm were ion-implanted with 1.2 keV ^32^S^+^ into a depth of approximately 40 nm at room temperature. The implantation doses were 1 × 10^14^, 1 × 10^15^, and 1 × 10^16^ ions/cm^2^. Pulsed laser melting (PLM) was carried out by 1 kHz train of 100 fs, 800 nm femtosecond laser pulses with a fluence of 0.5 J/cm^2^. Then, laser spot of 200 μm diameter is focused on the silicon and patterned square areas up to 10 mm × 10 mm. Rapid thermal annealing (RTA) was implemented at 600 °C for 30 min in a N_2_ atmosphere.

We determined the absorptance (*A*) of the samples by measuring reflectance (*R*) and transmittance (*T*) by using a UV-Vis-NIR spectrophotometer (UV3600, Shimadzu, Tokyo, Japan) equipped with an integrating sphere detector [3]. The absorptance was calculated by *A* = 1-*R*-*T*. The concentration and mobility of carriers were measured by Hall Effect measurement system at room temperature (via van der Pauw technique) [14]. To investigate whether the impurity/intermediate band (IB) formed by sulfur impurities in silicon enhances the sub-bandgap photoresponse, we employed a Fourier-transform photocurrent spectroscopy method as described in Ref. [15, 16], where the chopped FTIR globar light source is focused onto the sample, and the generated photocurrent is then demodulated by an external lock-in amplifier and finally fed back to the external port of the FTIR.

## Results and Discussion

**Fig. 1:**
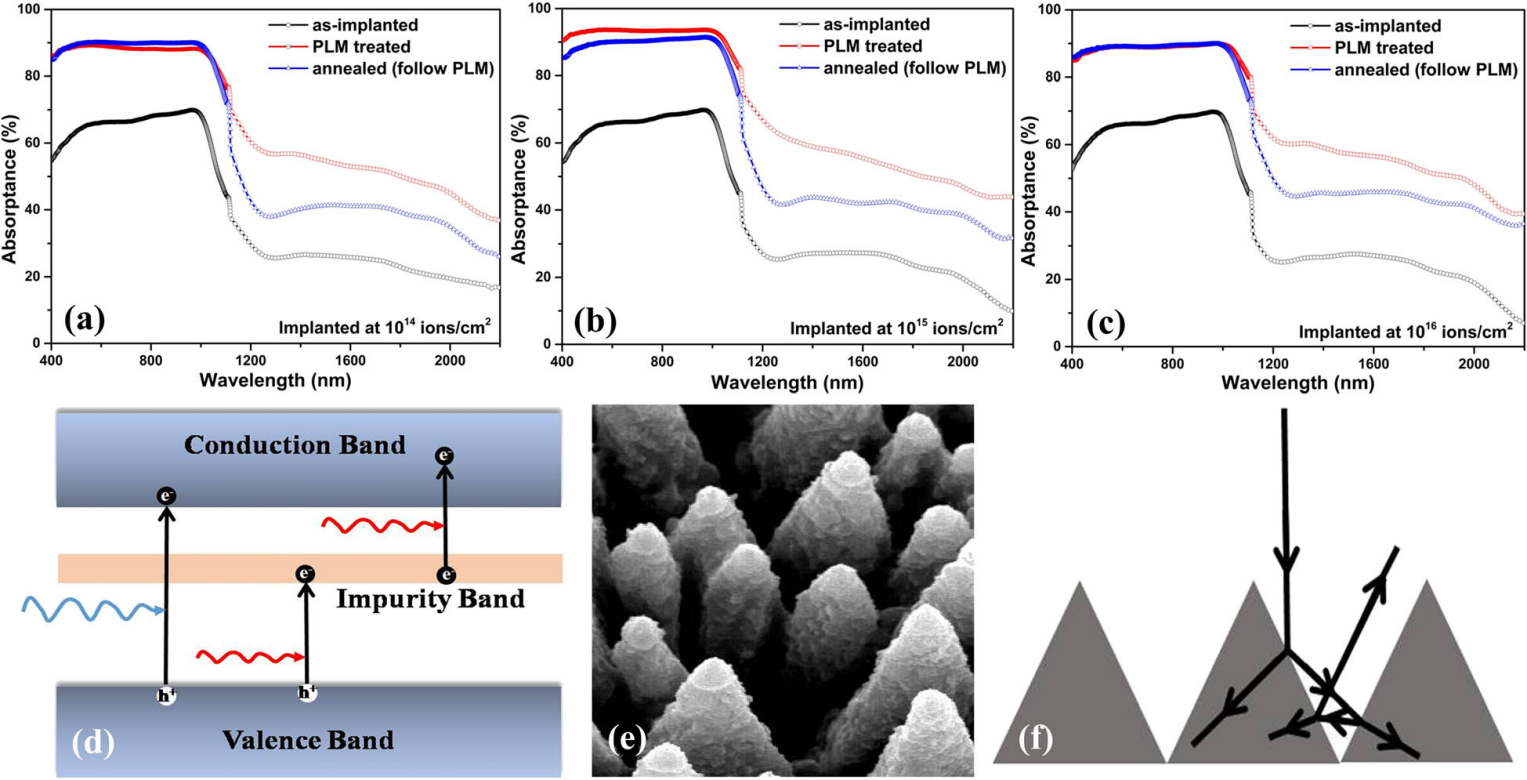
**a**–**c** Dependence of absorptance on different fabrication process with various implantation doses. **d** Impurity band located within bandgap of Si facilitates generation of carriers which participate in absorption of lower energy photons. **e** Scanning electron micrograph of silicon spikes. **f** Illustration of optical path on microstructured surface

Figure 1 shows the absorptance of silicon samples implanted at different doses. The samples processed with PLM showed highest absorptance at visible and NIR wavelengths while as-implanted samples showed lowest absorptance. However, the annealing process reduces the absorption in NIR region of spectra. The high Vis-NIR absorptance of microstructured silicon is ascribed to the following reasons: hyperdoping-induced impurity band and microstructured surface-generated light trapping effect. As illustrated in Fig. 1d, an impurity band induced by dopants is formed in silicon, which is responsible for sub-bandgap absorption [17]. Consequently, the hyperdoped silicon shows high absorptance in NIR range. Meanwhile, laser melting reconstructs the silicon surface and produces an array of cones that leads to multiple reflection and absorption [13], as displayed in Fig. 1e, f. The processed annealing evidently reduces absorptance at NIR wavelengths range, which mainly caused by the two aspects: (1) annihilate the nanostructures on the silicon surface, decreasing the light trapping effect [18]; and (2) result in the bond rearrangement within silicon matrix, which optically inactivate sulfur impurities [11].

**Fig. 2:**
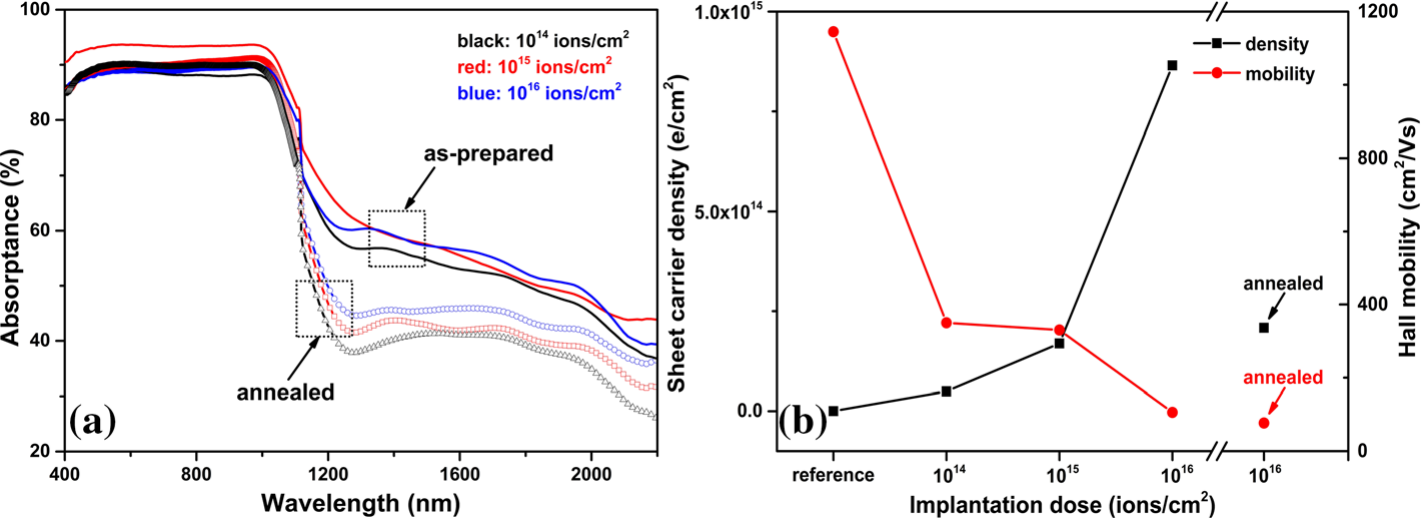
**a** Dependence of absorptance on different ion-implantation dose. All samples were microstructured by PLM. **b** Electronic properties of reference silicon and microstructured silicon for different ion-implantation dose before annealing and one after annealing

Because of the similar surface structure created by same laser parameters, the intensity of absorption in NIR range mainly depends on the dopant’s impurity levels [19]. In the past, we have illustrated the possible S-related energy levels corresponding to the photoresponse spectral features [20]. It showed the large enhancement observed at NIR region dependently resulted from the S-related energy level (~ 614 meV), which greatly enhanced the sub-bandgap absorptance. Prior to annealing process, absorption has no dramatic change with respect to the doping dose as shown in Fig. 2a. The microstructured silicon with 10^16^ and 10^15^ ion/cm^2^ implantation dose show similar absorptance, and the sample implanted at 10^14^ ions/cm^2^ shows unnoticeable decrease. We consider the lower absorptance for annealed samples in NIR range can be ascribed to the two aspects. M. A. Sheehy et al. [21] proposed the absorption decrease of below bandgap after annealing process is attributed to the diffusion out of the crystalline grains to the grain boundaries of the supersaturated dopants and defects. These defects include vacancies, dangling bonds, and floating bonds. Once the defects diffuse to the grain boundaries, they would no longer make a contribution to impurity bands in the Si, thus reducing the absorption of below bandgap radiation. Moreover, the literature [22] reported that no remarkable redistribution of S occurred until the annealing temperature reached at 650 °C. During this process, the S appears to complex with defect clusters, which means the S atoms will combine with each other at the Si wafer surface. This phenomenon leads to a reduction of the active doping concentration.

The carrier density and mobility of microstructured silicon with different ion-implantation doses are shown in Fig. 2b. It is evident that sheet density increases with ion-implantation dose, and mobility decreases with increasing ion-implantation dose. According to Shockley-Read-Hall (SRH) recombination effect, in an indirect bandgap semiconductor such as Si and Ge, the carrier lifetime decreases with the increase of dopant concentration [23, 24]. The decrease of mobility leads to an increase of recombination probability, so the decrease of mobility results in a decrease on electron lifetime and the decrease on mobility with increasing doping dose is consistent with SRH recombination effect. After annealing, the sheet carrier density decreases dramatically due to thermal diffusion effect as we discussed previously.

**Fig. 3:**
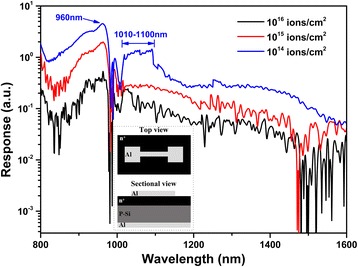
Photoresponse of n+/p detectors with different ion-implantation dose. Inset shows the top view and sectional view of the device. Light gray shows the patterns of interdigitated contact on microstructured surface and all standing contact on backside

Figure 3 shows the photoresponse with different doping dose, and the inset shows the diagram of n+/p photodetector. The photoresponse at NIR range indicates the appearance of impurity-mediated band. The prominent peak at approximately 960 nm corresponds to the generation of electron-hole pairs in silicon substrate, which are separated by the built-in potential of n^+^/p junction and collected at the top and bottom Al contacts. This phenomenon is well known as the heterojunction theory in Si devices [25].

The observed photoresponse in NIR is ascribed to the sulfur impurity levels in hyperdoped silicon. Such impurity levels facilitate the below bandgap absorption as mentioned above. The absorbed NIR light is converted into electron-hole pairs, resulting in the enhancement of photoresponse in NIR range (1100 ~ 1600 nm) [20]. The device with implantation dose of 10^14^ ions/cm^2^ shows the highest photoresponse in the wavelength range of 1010–1100 nm. The broad peak has been investigated owning to deep sulfur levels in femtosecond laser-processed silicon [20, 26]. In addition, we found that the device with 10^14^ ions/cm^2^ has showed higher photoresponse than those with 10^15^ and 10^16^ ions/cm^2^. And the Hall measurement indicated that the sample implanted at 10^14^ ions/cm^2^ had a bulk concentration of 10^19^ ions/cm^3^. As demonstrated by SRH recombination effect, carrier lifetime depends on dopant concentration in silicon. E. Mazur has concluded that the sample with 10^19^ ions/cm^3^ dopant concentration was expected to show longer carrier lifetime than 10^20^ and 10^21^ ions/cm^3^ [23]. Our Hall measurement results, sample implanted at 10^14^ ions/cm^2^ shows the highest mobility, are in agreement with the conclusion. Based on this theory, although a sample with higher doping dose shows greater absorptance, there is still a balance between optical absorption and carrier mobility. As presented in Fig. 3, the device with 10^14^ ions/cm^2^ is most probable to show the highest photoresponse, which is consistent with the conclusion reported in Ref. [23].

## Conclusions

We have measured the response of photodetectors based on microstructured silicon with different ion-implantation dose. The incorporation of impurities leads to a remarkable enhancement on absorptance and photoresponse at NIR wavelengths. And device implanted at 10^14^ ions/cm^2^ exhibits the highest photoresponse. PLM combined with ion-implantation demonstrates a considerable technique for the fabrication of NIR detectors. This technique may offer a feasible approach to fabricate low-cost broadband silicon-based photodetectors.

## References

[1] Wu C, Crouch CH, Zhao L (2001). Near-unity below-band-gap absorption by microstructured silicon. Appl Phys Lett.

[2] Jiang J, Li S, Jiang Y (2013). Mechanism of optical absorption enhancement of surface textured black silicon. J Mater Sci Mater Electron.

[3] Zhang T, Zhang P, Li S (2013). Black silicon with self-cleaning surface prepared by wetting processes. Nanoscale Res Lett.

[4] Zhang P, Li S, Liu C (2014). Near-infrared optical absorption enhanced in black silicon via Ag nanoparticle-induced localized surface plasmon. Nanoscale Res Lett.

[5] Su Y, Zhang P, Jiang J (2013). Absorption enhancement of near infrared in Te doped nanoporous silicon. J Mater Sci Mater Electron.

[6] Jiang J, Li S, Jiang Y (2012). Enhanced ultraviolet to near-infrared absorption by two-tier structured silicon formed by simple chemical etching. Philo Mag.

[7] Li S, Jiang Y, Wu Z (2011). Origins of 1/f noise in nanostructure inclusion polymorphous silicon films. Nanoscale Res Lett.

[8] Huang Z, Carey JE, Liu M (2006). Microstructured silicon photodetector. Appl Phys Lett.

[9] Yu P, Wu J, Liu S et al (2016) Design and fabrication of silicon nanowires towards efficient solar cells. Nano Today 11(6):704-737

[10] Crouch CH, Carey JE, Warrender JM (2004). Comparison of structure and properties of femtosecond and nanosecond laser-structured silicon. Appl Phys Lett.

[11] Crouch CH, Carey JE, Shen M (2004). Infrared absorption by sulfur-doped silicon formed by femtosecond laser irradiation. Appl Phys A Mater Sci Process.

[12] Carlson RO, Hall RN, Pell EM (1959). Sulfur in silicon. J Phys Chem Solids.

[13] Younkin RJ (2001). Surface studies and microstructure fabrication using femtosecond laser pulses.

[14] Tabbal M, Kim T, Warrender JM (2007). Formation of single crystal sulfur supersaturated silicon based junctions by pulsed laser melting. J Vac Sci Technol B Microelectron Nanometer Struct Process Meas Phenom.

[15] Vanecek M, Poruba A (2002). Fourier-transform photocurrent spectroscopy of microcrystalline silicon for solar cells. Appl Phys Lett.

[16] Liu Z, Kim JH, Fernandes GE (2009). Room temperature photocurrent response of PbS/InP heterojunction. Appl Phys Lett.

[17] Krich JJ, Halperin BI, Aspuru-Guzik A (2012). Nonradiative lifetimes in intermediate band photovoltaics—absence of lifetime recovery. J Appl Phys.

[18] Carey JE (2004). Femtosecond-laser microstructuring of silicon for novel optoelectronic devices.

[19] Janzén E, Stedman R, Grossmann G (1984). High-resolution studies of sulfur-and selenium-related donor centers in silicon. Phys Rev B.

[20] Zhang T, Ahmad W, Liu B (2017). Broadband infrared response of sulfur hyperdoped silicon under femtosecond laser irradiation. Mater Lett.

[21] Sheehy MA, Tull BR, Friend CM (2007). Chalcogen doping of silicon via intense femtosecond-laser irradiation. Mater Sci Eng B.

[22] Wilson RG (1984). Depth distributions of sulfur implanted into silicon as a function of ion energy, ion fluence, and anneal temperature. J Appl Phys.

[23] Mazur E, Franta B, Pastor D (2015). Laser doping and texturing of silicon for advanced optoelectronic devices. 2015 11th Conference on Lasers and Electro-Optics Pacific Rim (CLEO-PR). IEEE.

[24] Yakimov A, Kirienko V, Timofeev V (2014). Influence of delta-doping on the hole capture probability in Ge/Si quantum dot mid-infrared photodetectors. Nanoscale Res Lett.

[25] Pankove JI (1971). Optical processes in semiconductors.

[26] Guenther KM, Gimpel T, Tomm JW (2014). Excess carrier generation in femtosecond-laser processed sulfur doped silicon by means of sub-bandgap illumination. Appl Phys Lett.

